# Tanshinone IIA Ameliorates Nonalcoholic Steatohepatitis in Mice by Modulating Neutrophil Extracellular Traps and Hepatocyte Apoptosis

**DOI:** 10.1155/2022/5769350

**Published:** 2022-09-01

**Authors:** Lianjie Xu, Xiao Liu, Tao Jia, Yanhong Sun, Yan Du, Shanshan Wei, Wei Wang, Yurong Zhang, Wenhui Chen, Shan Zhang

**Affiliations:** ^1^Faculty of Basic Medicine, Yunnan University of Traditional Chinese Medicine, Kunming 650500, Yunnan, China; ^2^Qujing Hospital of Traditional Chinese Medicine, Qujing 655000, Yunnan, China; ^3^Department of Orthopedics, First Clinical Medical College of Yunnan University of Traditional Chinese Medicine, Kunming 650021, Yunnan, China; ^4^Yunnan Provincial Key Laboratory of Molecular Biology for Sinomedicine, Kunming 650500, Yunnan, China

## Abstract

*Salvia miltiorrhiza* Bunge, a traditional Chinese medicine, is widely used in the treatment of a variety of diseases and syndromes. Tanshinone IIA (TIIA), a phenanthrenequinone-class derivative extracted from *S. miltiorrhiza*, is one of its main active components and has anti-inflammatory effects on various tissues and cells. This study aimed to investigate the beneficial effects of TIIA on nonalcoholic steatohepatitis (NASH) induced in mice using a methionine choline deficiency (MCD) diet and the underlying mechanism of these. Our results reveal that TIIA remarkably ameliorated hepatic steatosis and inflammation and decreased the serum levels of liver dysfunction markers while increasing the levels of serum total cholesterol and triglycerides in MCD-fed mice. TIIA significantly reduced mRNA levels of the inflammatory factors TNF-*α*, IL-6, and TGF-*β*. Similarly, TIIA inhibited caspase-3 and Bax-mediated apoptosis in MCD-fed mice. Together, our data indicate that TIIA inhibits the formation of MPO and CitH3 in neutrophil extracellular traps and inhibits apoptosis mediated by caspase-3 and Bax in hepatocytes, thereby mitigating inflammatory progression in an MCD diet-induced NASH mouse model.

## 1. Introduction

Nonalcoholic fatty liver disease (NAFLD) is one of the most common liver diseases worldwide. Nonalcoholic steatohepatitis (NASH) is a progressive form of NAFLD that may progress to liver fibrosis, cirrhosis, and hepatocellular carcinoma (HCC) [1, 2]. The pathophysiology of NASH is complex and remains unclear.

Innate immune activation plays a key role in NASH pathogenesis [3]. Studies have demonstrated that neutrophils are pivotal protagonists in NASH, participating in the initiation and development of the disease [4]. In 2004, Brinkman et al. [5] discovered that activated neutrophils could also participate in the capture and killing of pathogens by releasing neutrophil extracellular traps (NETs). NETs are a form of extracellular reticular structure that can resist toxic factors and kill bacteria. They are composed of more than 30 proteins, including histones, neutrophil elastase (NE), myeloperoxidase (MPO), cathepsin G, and leukocyte protein 3 (PR3) [6]. Although NETs can kill bacteria, excessive NET formation leads to tissue deterioration as a result of inflammation [7]. Numerous studies have revealed the pivotal contribution of NETs and their components to NASH pathogenesis. Rensen et al. [8] found that MPO deficiency can significantly reduce the activation of hepatic stellate cells, fibrosis development, and accumulation of hepatocyte damage, suggesting that MPO plays an important role in NETs and promotes NASH pathogenesis and progression. Pulli et al. [9] studied a NASH model established using NE-knockout mice and wild-type mice. The results showed an increase in NE expression in the liver tissue of wild-type NASH mice, while NASH mice lacking NE displayed reduced body weight, reduced dyslipidaemia, and downregulated inflammation. NE can regulate ceramide metabolism in the liver in vivo and in vitro, suggesting that NETs play an important role in NASH by promoting inflammation. In addition, platelet-neutrophil interactions are important in NET-induced thrombosis, in both infectious and noninfectious diseases. Elevated levels of the MPODNA complex, a NET marker, have been found in blood serum of patients with NASH [10].

Furthermore, increased hepatocyte apoptosis has been observed in animal models and patients with NASH [11]. The main apoptotic pathways include death receptor activation and the mitochondrial and endoplasmic reticular pathways. These pathways can lead to hepatocyte apoptosis and contribute to NASH pathogenesis. The death receptor pathway is a TNF family-mediated apoptosis signalling pathway that activates caspase-8 and cleaves the active fragment tBid formed by bids, resulting in altered mitochondrial permeability [12]. This causes the release of cytochrome c and apoptosis-inducing factors, the activation of a caspase cascade involving caspase-3, caspase-6, and caspase-7, and, finally, apoptosis [12]. Alkhouri et al. [13] found that the death receptor apoptosis pathway in serum of patients with NASH was significantly overactive, suggesting that it is involved in NASH pathogenesis. Mitochondria are central to apoptosis. The mitochondrial pathway involves the proapoptotic proteins Bcl-2 and Bax, which play key roles in regulating hepatocyte apoptosis. Studies have shown ultrastructural mitochondrial damage in patients with NASH, and the deeper the lesion, the more obvious the functional damage [14]. Endoplasmic reticulum stress (ERS) is the core of the ERS pathway, which not only increases the expression of inflammatory factors but also triggers the phosphorylation of tumour necrosis factor receptor-associated factor 2, which leads to the activation of JNK and mediates apoptosis [15]. In recent years [16], apoptotic inhibitors have been developed to treat NASH. Therefore, inhibition of hepatocyte apoptosis and NET formation are promising for the diagnosis and treatment of NASH.

Tanshinone IIA (TIIA) is one of the main active components in *Salvia miltiorrhiza.* It has been reported to have antioxidant [17], anti-inflammatory [18], antiatherosclerotic [19], anticancer [20], and other pharmacological effects and has been widely used in the treatment of cardiovascular diseases [21]. Recent studies have demonstrated that TIIA-attenuated CCl_4_-induced liver injury and fibrosis inhibit the proliferation and activation of hepatic stellate cells (HSC) [22]. TIIA reduced liver injury induced by CCl_4_ combined with alcohol in a cirrhotic rat model by promoting the proliferation and differentiation of endogenous hepatic stem cells [23]. Furthermore, TIIA treatment can reduce the production of reactive oxygen species and malondialdehyde, reduce existing reactive oxygen species, and ameliorate steatosis [24]. However, whether TIIA can attenuate inflammation in NASH remains unclear. In this study, we aimed to investigate the beneficial effects of TIIA on NASH induced in C57BL/6 mice using a methionine choline deficiency (MCD) diet. Our findings offer insights into the therapeutic potential of TIIA for NASH.

## 2. Materials and Methods

### 2.1. Animals and Treatments

Pathogen-free 7-week-old female C57BL/6 mice (18–20 g) were purchased from the Experimental Animal Centre of Kunming Medical University (Kunming, Yunnan, China). Mice were acclimatised in the facility seven days before the start of the experiment under standard environmental conditions (23 ± 2°C, 12/12 h light/dark cycle with lights on at 08 : 00) and had ad libitum access to food and water. All animals received humane care according to the institutional animal care guidelines approved by the Experimental Animal Ethics Committee of Kunming Medical University. Mice were randomly divided into three groups (*n* = 8 per group). The control group was continuously fed standard chow, the NASH group was fed MCD, and the NASH + TIIA group was fed the MCD diet (A02082002B, Research Diets) (composition: sucrose 455.3 g/kg, L-amino acids 171.4 g/kg, corn starch 150 g/kg, corn oil 100 g/kg, maltodextrin 50 g/kg, mineral mix 35 g/kg, cellulose 30 g/kg, sodium bicarbonate 7.5 g/kg, and vitamin mix 10 g/kg). Mice in the drug treatment group were intraperitoneally injected with TIIA at 30 mg/kg body weight per day during MCD diet feeding and were weighed daily. The mice in the remaining groups were intraperitoneally injected with an equal volume of normal saline. The doses of TIIA were selected based on previous studies in mice. At the end of the treatment period, the eyeballs were extracted for blood collection. The serum was collected by centrifugation, and the liver tissue was stored at −80°C for cryopreservation or fixed in 4% paraformaldehyde until subsequent use.

### 2.2. Reagents

Antimyeloperoxidase, anti-Bax, anticitrullinated histone 3, and anti-caspase-3 were purchased from Abcam (Cambridge, MA, USA); anti-caspase-9 was purchased from Proteintech (Proteintech Group, Inc., USA); and *β*-actin antibody was purchased from Cell Signaling Technology (Danvers, Massachusetts, USA); TRIzol was purchased from Invitrogen (Waltham, Massachusetts, USA); PrimeScipt™ RTMasterMix and SYBRPremixEX™II were purchased from TaKaRa (Japan), and sulfotanshinone sodium injection. Sangon Biotech (Shanghai, China) synthesized the primers for synthesis.

### 2.3. Biochemical Analysis

Serum aspartate aminotransferase (AST), alanine aminotransferase (ALT), triglyceride (TG), total cholesterol (TC), and total bilirubin levels were measured using commercial assays with an automated analyser (Roche Diagnostics, Mannheim, Germany).

### 2.4. Histopathology

Liver samples were collected from each mouse and fixed in 4% paraformaldehyde for at least 24 h at room temperature, embedded in paraffin, sectioned into 5 *μ*m tissues, and then stained with haematoxylin-eosin. Lipid accumulation in the liver was evaluated using Oil Red O (ORO) staining. Frozen sections were washed twice with PBS, then washed with 60% isopropanol for 1 min, dried at room temperature, stained with diluted ORO for 30 min, washed with distilled water and PBS for 5 min each, and sealed. Finally, the sections were washed with running water and mounted using glycerin buffer. Sections were photographed using an Axioscope microscope (Carl Zeiss, Germany).

### 2.5. Immunohistochemistry

Mouse liver tissues were fixed in 4% paraformaldehyde and embedded in paraffin. Liver tissue (5 *μ*m thick) was cut for immunohistochemical analysis. The tissue sections were dewaxed with xylene and dehydrated with ethanol. After immersion in a citric acid antigen repair solution at a pH of 6.0, the sections were washed with PBS. The slices were immersed in a 3% H_2_O_2_ solution at room temperature for 15 min to block endogenous peroxidase activity. The sections were then incubated with anti-MPO (1 : 40) overnight at 4°C. HRP-conjugated anti-rabbit secondary antibody was used during the first incubation, and the sections were then stained with 3,3′-diaminobenzidine (DAB), the substrate of chromogenic peroxidase. Last, the sections were stained with haematoxylin and examined under a light microscope.

### 2.6. MPO Activity Determination

Liver MPO activity was determined using an MPO test kit purchased from Nanjing Jiancheng Bioengineering Institute (Nanjing, China, A044-1-1). Frozen liver tissue was added nine times to normal saline ice homogenate, centrifuged at 3,000 r/min for 10 min, and colorimetry was performed according to kit instructions to detect MPO activity.

### 2.7. TUNEL Staining

Hepatocyte apoptosis was detected using a TUNEL Assay kit purchased from the Beyotime Institute of Biotechnology (Shanghai, China, C1091), according to the manufacturer's protocol. Briefly, paraffinized sections of liver tissue were dewaxed and hydrated, and protease K solution was added to remove tissue protein. Next, buffer containing 3% H_2_O_2_ was added. The reaction was carried out at room temperature for 20 min. The TDT enzyme reaction solution was added dropwise, and the reaction was carried out at 37°C for 1 h. Samples were incubated with streptavidin-horseradish peroxidase (HRP) at 37°C for 30 min. DAB staining, haematoxylin restaining, dehydration, and transparent sealing of the brain slides were examined microscopically. For TUNEL staining of apoptotic cell count, three sections of each animal's liver were randomly taken, and the tissue-rich area was selected from each section using a light microscope at 400-fold magnification. Apoptotic cells appeared brownish-yellow in colour. The proportion of apoptotic cells was calculated by dividing the number of apoptotic cells by the total number of cells.

### 2.8. Quantitative Reverse Transcription Polymerase Chain Reaction

A quantitative reverse transcription polymerase chain reaction (RT-qPCR) was used to determine the expression of genes involved in inflammation. Total RNA from liver tissue was extracted using TRIzol (Invitrogen) according to the manufacturer's instructions. Approximately 1 *μ*g of RNA was reverse-transcribed into cDNA using the PrimeScript RT Reagent kit (TaKaRa, Japan). RT-qPCR was performed in a Roche analyser using 96-well reaction plates, pairs of oligonucleotide primers, and TB green Premix EX Taq™ II for the mouse tumour necrosis factor (TNF)-*α* (sense: ACTGGCAGAAGAGGCACTCC, antisense: GCCACAAGCAGGAATGAGAA), interleukin (IL) 6 (sense: TCCATCCAGTTGCCTTCTTG, antisense: AAGCCTCCGACTTGTGAAGTG), transforming growth factor (TGF)-*β* (sense: GCAACAATTCCTGGCGTTACCTTG, antisense: CCTGTATTCCGTCTCCTTGGTTCAG), and *β*-actin (sense: TATCGGACGCCTGGTTA, antisense: TGTGCCGTTGAACTTGC). The relative expression level of mRNA was calculated using 2^ΔΔct^. The primers for the target genes were synthesised by Sangon Biotech Co., Ltd. (Shanghai, China).

### 2.9. Western Blotting

Liver proteins were extracted using RIPA Lysis Buffer (Beyotime, China) and 100 mM phenylmethanesulfonyl fluoride (Beyotime). The supernatant was collected via centrifugation at 4°C for 5 min. The protein samples were electrophoretically separated using sodium dodecyl sulfate-polyacrylamide gel electrophoresis (SDS-PAGE) and transferred to polyvinylidene difluoride membranes (PVDF) membranes. Membranes were incubated with primary antibodies (caspase-3, CitH3, Bax, and caspase-9) at 4°C overnight. After washing three times with TBST, the membranes were incubated with secondary antibodies for 1 h at room temperature. Proteins were detected using an enhanced chemiluminescence (ECL) reagent. Following another triple wash, proteins were visualised using a chemiluminescence system. The intensities of protein bands in the Western blot were quantified using the ImageJ software.

### 2.10. Double-Labelled Immunofluorescence

For immunofluorescent staining of paraffin sections, 5 *μ*m-thick liver tissue sections were deparaffinized with xylene and rehydrated with ethanol and water. After immersion in citric acid antigen repair solution (pH 6.0), the sections were washed with PBS. The slices were immersed in 3% H_2_O_2_ solution at room temperature for 15 min to block endogenous peroxidase activity. The liver tissues were incubated with primary antibodies against anti-MPO and CitH3 and fluorescent secondary antibodies 488-goat anti-rabbit and cy3-goat anti-rabbit. Sections were observed under a Nikon upright fluorescence microscope. Nuclei stained by DAPI were blue upon excitation with ultraviolet light, and positive expression was determined by observing the corresponding fluorescein-labelled red or green light.

### 2.11. Statistical Analysis

Result analysis and mapping were performed using SPSS 16.0 and GraphPad Prism 5.0, and the experimental data were expressed as mean ± standard deviation (S.D.). One-way analysis of variance was used to compare multiple groups, and Dunnett's test was used to compare two samples. Differences were considered statistically significant at *P* < 0.05.

## 3. Results

### 3.1. Effects of TIIA on Body Weight, Food Intake, and Liver Weight in Mice with NASH Induced by MCD Diet

**Figure 1:**
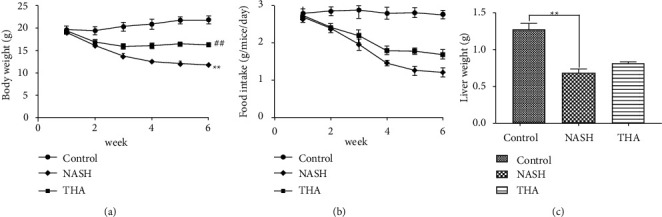
Effects of TIIA on body weight, food intake, liver weight, and liver index in mice with NASH induced by MCD diet. (a) Body weight in the control and NASH groups differed significantly from week 2 to week 6 (^*∗∗*^*P* < 0.01, *n* = 8). The body weight of TIIA mice increased from week 3 to week 6 when compared to NASH (^**##**^*P* < 0.01, *n* = 8). (b) Control mice maintained stable food intake during the modelling period. From the third week, the food intake of TIIA mice was higher than that of NASH mice. (c) Compared to the control group, the liver weight of the NASH group decreased (^*∗∗*^*P* < 0.01, *n* = 8). Compared with the NASH group, the liver weight of the TIIA group increased, but the difference was not statistically significant.

To explore the effect of TIIA in the treatment of NASH, C57/BL6 mice were fed a normal or MCD diet for up to six weeks, and changes in body weight, food intake, and liver weight were assessed. Compared to the control group, the body weight of mice in the NASH group decreased between 2 and 6 weeks. Compared to the NASH group, the body weight of mice in the TIIA group increased at 3–6 weeks (Figure 1(a)). The food intake of mice in the control group remained stable during the modelling period, while the food intake of mice in the NASH and TIIA groups showed a downward trend (Figure 1(b)). Beginning in the third week, the food intake of mice in the TIIA group was higher than that of mice in the NASH group (Figure 1(b)). Moreover, we found that MCD-fed mice displayed a decrease in liver weight and that liver weight increased after TIIA treatment, but the difference was not statistically significant (Figure 1(c)).

### 3.2. TIIA Attenuates Liver Dysfunction Status in MCD-Induced NASH

**Figure 2:**
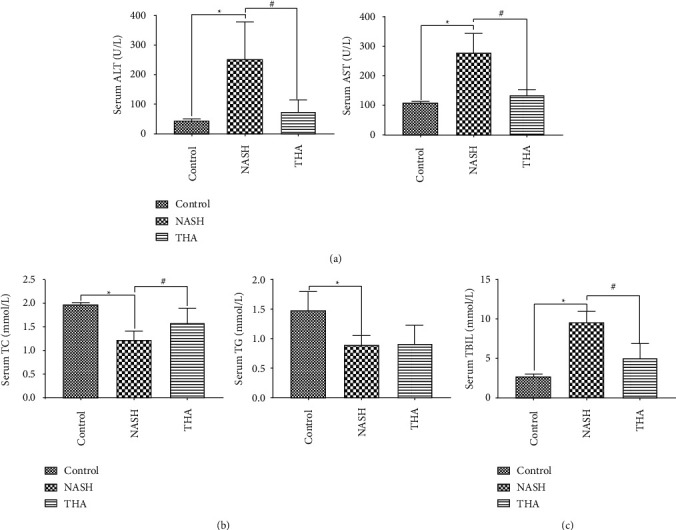
Effects of TIIA on serum transaminase, total bilirubin, and lipid levels in MCD-fed mice. (a) Serum transaminase level in mice (ALT and AST). (b) Serum cholesterol and triglyceride levels. (c) Total serum bilirubin. ^*∗*^*P* < 0.05, when compared to the control; ^#^*P* < 0.05, when compared to NASH, *n* = 8.

Mice fed the MCD diet developed steatohepatitis, similar to the histology of NASH liver disease. We found that MCD induced a significant increase in the activity of ALT and AST (Figure 2(a)), which are markers of liver dysfunction. TIIA treatment significantly altered the activity of ALT and AST, which completely prevented the increase in liver transaminase levels in MCD-fed mice (Figure 2(a)). In addition, when compared to the control group, the MCD diet significantly reduced serum triglyceride and total cholesterol levels in NASH mice, and the serum TC and TG levels showed an upward trend after TIIA treatment (Figure 2(b)). Compared to the control group, the serum bilirubin levels of NASH mice increased and total bilirubin levels decreased after TIIA treatment (Figure 2(c)). Collectively, these data support the idea that TIIA has beneficial effects on liver function and blood cholesterol status.

### 3.3. TIIA Alleviates Liver Steatosis and Inflammation in MCD-Induced NASH

**Figure 3:**
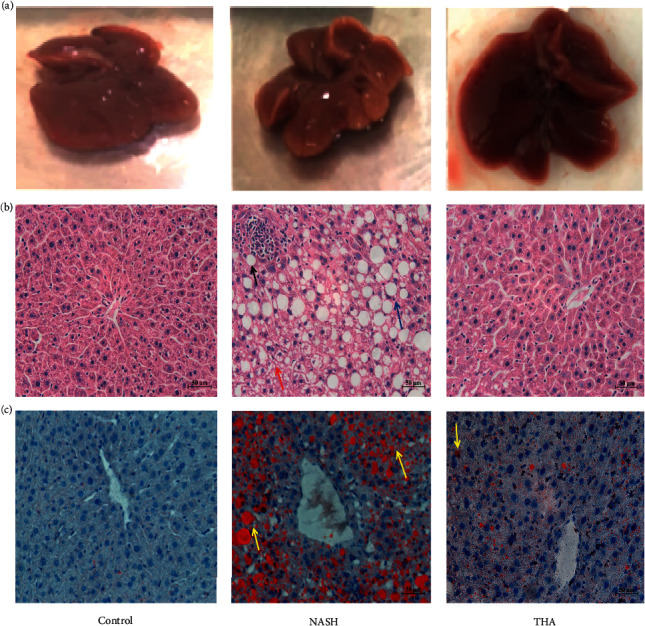
TIIA alleviated liver steatosis and inflammation in MCD-induced NASH. (a) Representative macroscopic appearance of livers. (b) H&E staining of liver sections. The black arrow indicates inflammatory cell infiltration, the red arrow indicates loose foreskin, and the yellow arrow indicates steatosis. Original magnification: x400. (c) Oil Red O staining of liver sections from mice. Yellow arrows refer to grease droplets. Original magnification: x400.

We observed that after 6 weeks of an MCD diet, the liver tissue of the model mice was pale yellow and had a reduced volume when compared to the liver tissue of control mice. The liver appeared dark red after TIIA treatment (Figure 3(a)). H&E staining showed hepatic steatosis, ballooning, and inflammatory cell infiltration in MCD-induced NASH mice when compared to normal mice. TIIA treatment significantly reduced steatosis in mice (Figure 3(b)). Oil Red O staining results showed that a large number of red lipid droplets accumulated in the NASH group, and the number of droplets decreased significantly after TIIA treatment (Figure 3(c)). Taken together, histological analysis showed that TIIA significantly reduced steatosis, ballooning degeneration, inflammatory infiltration, and lipid deposition in NASH mice.

### 3.4. TIIA Modulates Hepatic Expression of Inflammation in MCD-Induced NASH

**Figure 4:**
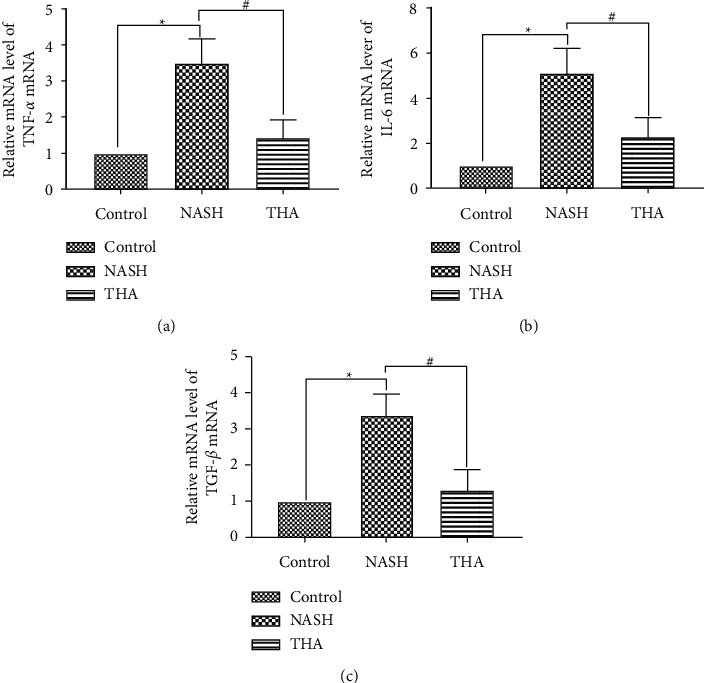
Effect of TIIA on the mRNA levels of inflammatory factors in MCD-fed mice. (a) Quantitative real-time polymerase chain reaction (RT-qPCR) analyses of TNF-*α*, (b) IL-6, and (c) TGF-*β*. ^*∗*^*P* < 0.05 when compared to the control; ^#^*P* < 0.05 when compared to NASH, *n* = 8.

In recent years, studies have found that a variety of inflammatory factors and adipocytokines, including TNF-*α* and IL-6, play a role in NASH pathogenesis. High expression of TNF-*α* and IL-6 [25] was detected in patients with NASH, which has been shown to play a key role in NASH pathogenesis [26]. TNF-*α* can activate IL-6, IL-8, and other cytokines, form an inflammatory cascade, induce liver inflammation, lead to insulin resistance (IR), and cause NASH [27]. We performed RT-qPCR to determine whether TIIA could reduce the expression of TNF-*α* and IL-6 in NASH mice. Compared to the control group, the mRNA levels of the proinflammatory cytokines TNF-*α* and IL-6 were significantly increased in the liver tissue of MCD-fed mice. The mRNA levels of TNF-*α* and IL-6 were significantly reduced by TIIA treatment (Figure 4(a) and 4(b)). Moreover, fibrosis is one of the pathological features of NASH, and its progression can also be regarded as the progression of inflammation. TGF-*β* is an important regulatory factor in liver fibrosis. To explore whether TIIA can inhibit fibrosis progression, TGF-*β* expression was analysed using RT-qPCR. The results showed that TIIA treatment reduced TGF-*β* mRNA levels (Figure 4(c)).

### 3.5. TIIA Inhibited the Formation of MPO and CitH3

**Figure 5:**
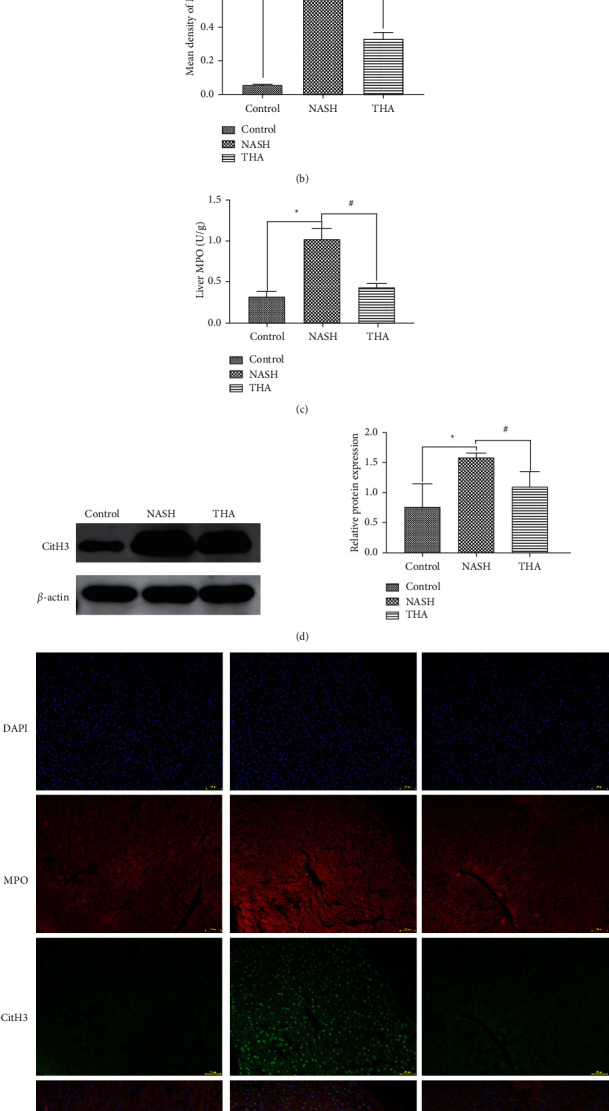
TIIA inhibited the formation of MPO and CitH3. (a) Immunohistochemical analyses of liver myeloperoxidase (MPO). Original magnification: x400. Arrows indicate positive cells. (b) The staining signal intensity analysed using Image-Pro Plus. (c) The liver tissues of different groups analysed for MPO expression using a test kit. (d) The liver tissues of different groups analysed for CitH3 protein expression via Western blotting. (e) The MPO and CitH3 expressed in NASH labelled by fluorescent double labelling. Original magnification: x400. ^*∗*^*P* < 0.05  when compared to the control; ^#^*P* < 0.05 when compared to NASH, *n* = 3.

We performed immunohistochemical analysis to investigate whether TIIA reduces MPO expression in NASH mice. Compared to the control group, the expression of MPO in the NASH group was significantly increased. However, TIIA significantly inhibited MPO upregulation (Figures 5(a) and 5(b)). MPO test kit analysis showed that TIIA treatment reduced MPO expression in the livers of NASH mice (Figure 5(c)). Western blot analysis showed that TIIA treatment reduced CitH3 expression in the livers of NASH mice (Figure 5(d)), demonstrating the efficacy of TIIA in the inhibition of NET formation. In addition, using an immunofluorescence assay, we found that TIIA inhibited the expression of MPO and CitH3 (Figure 5(e)).

### 3.6. TIIA Modulates Hepatic Expression of Apoptotic Mediators in MCD-Induced NASH

**Figure 6:**
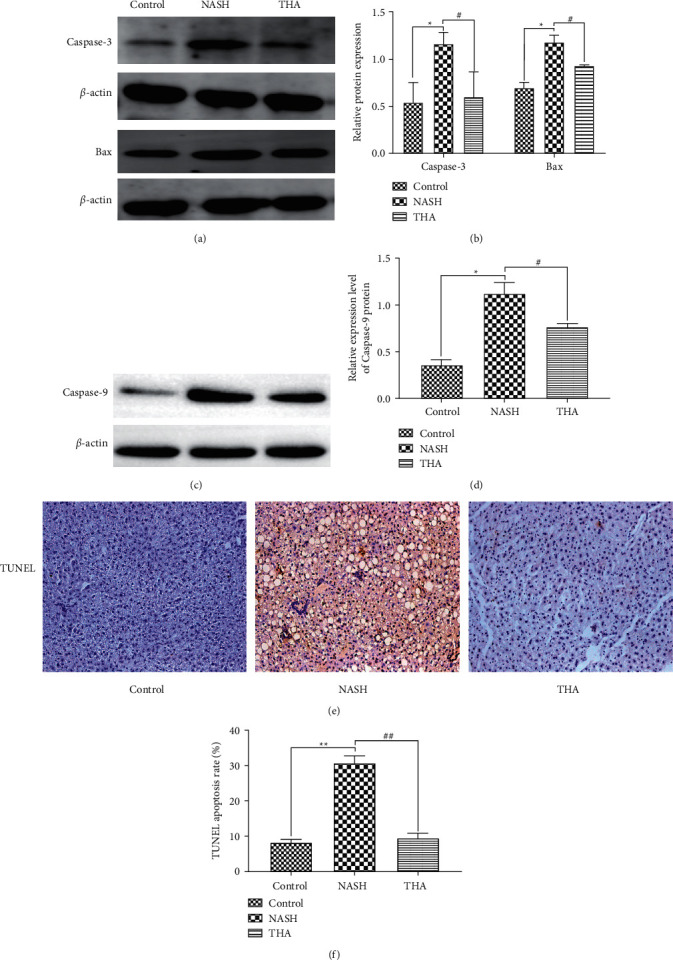
TIIA inhibits hepatocyte apoptosis. (a) Western blot analysis of liver Bax and caspase-3. Results were normalised relative to the expression of *β*-actin. (b) Density analyses of caspase-3 and Bax expression. ^*∗*^*P* < 0.05 when compared to the control; ^#^*P* < 0.05 when compared to NASH, *n* = 3. (c) Western blot analysis of liver caspase-9. Results were normalised relative to the expression of *β*-actin. (d) Density analyses of caspase-9 expressions. ^*∗*^*P* < 0.05 compared to the control; ^***#***^*P* < 0.05 compared to NASH, *n* = 3. (e) TUNEL analysis of hepatocyte apoptosis. Original magnification: x200. (f) Apoptosis rate in each group. ^*∗∗*^*P* < 0.01 when compared to the control; ^##^*P* < 0.01 compared to NASH, *n* = 3.

Liver tissue apoptosis plays an important role in the occurrence and treatment of liver diseases [11]. Caspase-3, caspase-9, and Bax promote hepatocyte apoptosis. To investigate the effect of TIIA on hepatocyte apoptosis, we examined the expression levels of caspase-3, caspase-9, and Bax via Western blotting. Compared to the control group, the expression of caspase-3, caspase-9, and Bax in the NASH group was significantly increased. TIIA reduced caspase-3, caspase-9, and Bax expression and inhibited hepatocyte apoptosis (Figures 6(a)–6(d)). Hepatocyte apoptosis was also measured using the TUNEL assay and cell count. Compared to the control group, the number of TUNEL-positive cells in the NASH group was significantly increased, and TIIA treatment decreased the number of TUNEL-positive cells in the liver (Figures 6(e) and 6(f)).

## 4. Discussion

In this study, we provided evidence regarding the potent role of TIIA in reducing the severity of liver steatosis, inflammation, and fibrosis in an MCD-fed NASH model. Moreover, our results show that the protective effect of TIIA involves the inhibition of hepatocyte apoptosis and NET formation.

IL-1*β*, IL-6, and TNF-*α* are inflammatory markers. The expression levels of TNF-*α* and IL-6 are increased in serum of NASH animal models and patients [26]. TNF-*α* is one of the main proinflammatory cytokines that regulates innate and adaptive immune responses. It can inhibit the transcription of adiponectin in adipocytes and promote inflammation progression. IL-6 is a proinflammatory cytokine that plays a key role in local and systemic proinflammatory responses [28]. Our study shows that TIIA significantly inhibits the expression of proinflammatory cytokines TNF-*α* and IL-6 in NASH mice. Furthermore, with a decrease in liver inflammation, the serum levels of ALT, AST, and TBIL in TIIA-treated NASH mice were significantly reduced, which represents a reduction in liver injury. Moreover, TGF-*β* promotes NASH development through Smad signalling and ROS production, leading to hepatocyte death and lipid accumulation [29]. TIIA treatment also significantly inhibited TGF-*β* expression in NASH mice.

Neutrophils have long been noted in NASH inflammatory cell infiltrates. Neutrophils are innate immune cells that kill and capture bacteria through phagocytosis, degranulation, and NETs. NETs are extracellular structures of histone 3, neutrophil elastase, and myeloperoxidase [5]. Although the components of NETs have bactericidal activity, the excessive NET formation has a proinflammatory effect, leading to host cell damage [30]. CitH3 and MPO are NET components, and MPO and CitH3 are key steps in NET synthesis. MPO relies on NE to enter the nucleus, which promotes further depolymerisation of chromatin and degradation of the nuclear membrane [31, 32]. Depolymerised chromatin is released into the cytoplasm to form NETs. In addition, protein arginine deaminase type 4 (PAD4) promotes histone hypercitrullination to generate CitH3, which promotes chromatin decondensation to form NETs [33]. In our study, we found that TIIA significantly inhibits NET formation and the release of NETs associated with MPO and CitH3 in the TIIA-treated NASH group.

Hepatocyte apoptosis directly or indirectly promotes the progression of inflammation and fibrosis [34] and is the most common type of cell death in NASH [35]. The activation of caspases and Bcl-2 family proteins induces hepatocyte apoptosis and plays a role in NAFLD/NASH activation. Caspase-3 promotes apoptosis and is an important member of the caspase family. Clinical studies [36] have shown that the expression of caspase-3 in liver biopsy specimens of patients with NASH is significantly increased, and the expression of caspase-3 increases with disease progression. Animal experiments [37] have shown that in a diet-induced NASH model, caspase-3 inactivation has a protective effect on hepatocytes and can slow the development of fibrosis. Therefore, the inhibition of caspases can be regarded as a target of NASH drug therapy. As an important proapoptotic gene of the Bcl-2 family, the expression of Bax in the NASH group mice was higher than that in control group mice. Bax inhibitor-1 can prevent NASH by inhibiting IRE1*α* [38]. Our study also shows that TIIA treatment inhibits hepatocyte apoptosis by inhibiting the expression of caspase-3 and Bax in NASH mice.

## 5. Conclusions

In conclusion, our study shows that TIIA can ameliorate inflammation and reduce hepatocyte apoptosis in NASH mice by inhibiting NET formation associated with MPO and CitH3 release, thereby reducing TNF-*α*, IL-6, and TGF-*β* expression. These results suggest the potential applicability of TIIA as a therapeutic agent for NASH treatment.

## Data Availability

The data used to support this study are available from the corresponding author upon request.

## References

[1] Younossi Z., Anstee Q. M., Marietti M. (2018). Global burden of NAFLD and NASH: trends, predictions, risk factors and prevention. *Nature Reviews Gastroenterology & Hepatology*.

[2] Younossi Z. M., Loomba R., Rinella M. E. (2018). Current and future therapeutic regimens for nonalcoholic fatty liver disease and nonalcoholic steatohepatitis. *Hepatology*.

[3] Parthasarathy G., Revelo X., Malhi H. (2020). Pathogenesis of nonalcoholic steatohepatitis: an overview. *Hepatology Communications*.

[4] Wu L., Gao X., Guo Q. (2020). The role of neutrophils in innate immunity-driven nonalcoholic steatohepatitis: lessons learned and future promise. *Hepatology International*.

[5] Brinkmann V., Reichard U., Goosmann C. (2004). Neutrophil extracellular traps kill bacteria. *Science*.

[6] Vorobjeva N. (2020). Neutrophil Extracellular Traps: New Aspects. *Moscow University Biological Sciences Bulletin*.

[7] Papayannopoulos V. (2018). Neutrophil extracellular traps in immunity and disease. *Nature Reviews Immunology*.

[8] Rensen S. S., Bieghs V., Xanthoulea S. (2012). Neutrophil-derived myeloperoxidase aggravates non-alcoholic steatohepatitis in low-density lipoprotein receptor-deficient mice. *PLoS One*.

[9] Pulli B., Ali M., Iwamoto Y. (2015). Myeloperoxidase-hepatocyte-stellate cell cross talk promotes hepatocyte injury and fibrosis in experimental nonalcoholic steatohepatitis. *Antioxidants and Redox Signaling*.

[10] Arelaki S., Koletsa T., Sinakos E. (2022). *Neutrophil Extracellular Traps Enriched with IL-1β and IL-17A Participate in the Hepatic Inflammatory Process of Patients with Non-alcoholic Steatohepatitis*.

[11] Kanda T., Matsuoka S., Yamazaki M. (2018). Apoptosis and non-alcoholic fatty liver diseases. *World Journal of Gastroenterology*.

[12] Eugenia G. M., Harmeet M., Mott J. L., Gores G. J. (2013). Apoptosis and necrosis in the liver. *Comprehensive Physiology*.

[13] Alkhouri N., Alisi A., Okwu V. (2015). Circulating soluble fas and fas ligand levels are elevated in children with nonalcoholic steatohepatitis. *Digestive Diseases and Sciences*.

[14] García-Ruiz C., Baulies A., Mari M., García-Rovés P. M., Fernandez-Checa J. C. (2013). Mitochondrial dysfunction in non-alcoholic fatty liver disease and insulin resistance: cause or consequence?. *Free Radical Research*.

[15] Hu P., Han Z., Couvillon A. D., Kaufman R. J., Exton J. H. (2006). Autocrine tumor necrosis factor Alpha links endoplasmic reticulum stress to the membrane death receptor pathway through ire1*α*-mediated NF-*κ*B activation and down-regulation of TRAF2 expression. *Molecular and Cellular Biology*.

[16] Younossi Z. M., Stepanova M., Lawitz E. (2018). Improvement of hepatic fibrosis and patient-reported outcomes in non-alcoholic steatohepatitis treated with selonsertib. *Liver International*.

[17] Gong G., Gu Y., Zhang Y., Liu W., Li L., Li J. (2019). RETRACTED: tanshinone IIA alleviates oxidative damage after spinal cord injury in vitro and in vivo through up-regulating miR-124. *Life Sciences*.

[18] Fu K., Feng C., Shao L., Mei L., Cao R. (2021). Tanshinone IIA exhibits anti-inflammatory and antioxidative effects in LPS-stimulated bovine endometrial epithelial cells by activating the Nrf2 signaling pathway. *Research in Veterinary Science*.

[19] Tan Y. l., Ou H. x., Zhang M. (2019). Tanshinone IIA promotes macrophage cholesterol efflux and attenuates atherosclerosis of apoE^−/−^ mice by omentin-1/ABCA1 pathway. *Current Pharmaceutical Biotechnology*.

[20] Fang Z. Y., Zhang M., Liu J. N., Zhao X., Zhang Y. Q., Fang L. (2020). Tanshinone IIA: a review of its anticancer effects. *Frontiers in Pharmacology*.

[21] Ansari M. A., Khan F. B., Safdari H. A. (2021). Prospective therapeutic potential of Tanshinone IIA: an updated overview. *Pharmacological Research*.

[22] Shi M. J., Yan X. L., Dong B. S., Yang W. N., Su S. B., Zhang H. (2020). A network pharmacology approach to investigating the mechanism of Tanshinone IIA for the treatment of liver fibrosis. *Journal of Ethnopharmacology*.

[23] Yang N., Chen H., Gao Y. (2020). Tanshinone IIA exerts therapeutic effects by acting on endogenous stem cells in rats with liver cirrhosis. *Biomedicine & Pharmacotherapy*.

[24] Zhang S., Huang G., Yuan K. (2017). Tanshinone IIA ameliorates chronic arthritis in mice by modulating neutrophil activities. *Clinical and Experimental Immunology*.

[25] Polyzos S. A., Kountouras J., Polymerou V., Papadimitriou K. G., Zavos C., Katsinelos P. (2016). Vaspin, resistin, retinol-binding protein-4, interleukin-1*α* and interleukin-6 in patients with nonalcoholic fatty liver disease. *Annals of Hepatology*.

[26] Crespo J., Cayón A., Fernández-Gil P. (2001). Gene expression of tumor necrosis factor [alpha] and TNF-receptors, p55 and p75, in nonalcoholic steatohepatitis patients. *Hepatology*.

[27] Ruan H., Lodish H. (2003). Insulin resistance in adipose tissue: direct and indirect effects of tumor necrosis factor-alpha. *Cytokine Growth Factor Reviews*.

[28] Fernando M. R., Reyes J. L., Iannuzzi J., Leung G., McKay D. M. (2014). The pro-inflammatory cytokine, interleukin-6, enhances the polarization of alternatively activated macrophages. *PLoS One*.

[29] Yang L., Roh Y. S., Song J. (2014). Transforming growth factor beta signaling in hepatocytes participates in steatohepatitis through regulation of cell death and lipid metabolism in mice. *Hepatology*.

[30] Jorch S. K., Kubes P. (2017). An emerging role for neutrophil extracellular traps in noninfectious disease. *Nature Medicine*.

[31] Metzler K., Goosmann C., Lubojemska A., Zychlinsky A., Papayannopoulos V. (2014). A myeloperoxidase-containing complex regulates neutrophil elastase release and actin dynamics during NETosis. *Cell Reports*.

[32] Papayannopoulos V., Metzler K. D., Hakkim A., Zychlinsky A. (2010). Neutrophil elastase and myeloperoxidase regulate the formation of neutrophil extracellular traps. *Journal of Cell Biology*.

[33] Wang Y., Li M., Stadler S. (2009). Histone hypercitrullination mediates chromatin decondensation and neutrophil extracellular trap formation. *Journal of Cell Biology*.

[34] Canbay A., Kip S. N., Kahraman A., Gieseler R. K., Nayci A., Gerken G. (2005). Apoptosis and fibrosis in non-alcoholic fatty liver disease. *Turkish Journal of Gastroenterology*.

[35] Wang L., Lv Y., Yao H., Yin L., Shang J. (2015). Curcumin prevents the non-alcoholic fatty hepatitis via mitochondria protection and apoptosis reduction. *International Journal of Clinical and Experimental Pathology*.

[36] Ferreira D. M. S., Castro R. E., Machado M. V. (2011). Apoptosis and insulin resistance in liver and peripheral tissues of morbidly obese patients is associated with different stages of non-alcoholic fatty liver disease. *Diabetologia*.

[37] Thapaliya S., Wree A., Povero D. (2014). Caspase 3 inactivation protects against hepatic cell death and ameliorates fibrogenesis in a diet-induced NASH model. *Digestive Diseases and Sciences*.

[38] Lebeaupin C., Vallée D., Rousseau D. (2018). Bax inhibitor-1 protects from nonalcoholic steatohepatitis by limiting inositol-requiring enzyme 1 alpha signaling in mice. *Hepatology*.

